# Hard Rubber and Corundum Disks and Wheels

**Published:** 1887-10

**Authors:** Geo. A. Maxfield

**Affiliations:** Holyoke, Mass.


### HARD RUBBER AND CORUNDUM DISKS AND WHEELS.

READ BEFORE THE CONNECTICUT VALLEY DENTAL SOCIETY, AT MONTREAL, JULY 21, 1887.

BY GEO. A. MAXFIELD, D. D. S., HOLYOKE, MASS.

Among the many appliances that have been brought to the notice of the dental practitioner within the past few years, none have proved of greater utility than corundum disks and wheels. We are indebted to Dr. Arthur for the introduction of these implements, and although the object for which he advocated their use —the making of permanent separations between the teeth—is justly condemned on all sides, the utility of the disks for other purposes is so great, that they will ever remain as part of the necessary equipment of the dental engine. What is known as the Arthur disks and wheels, is a combination of corundum or emery with shellac. Their softness, which causes them to wear away very rapidly, is one great objection to their use. To overcome this difficulty, experiments have been made with other materials, such as celluloid, gutta-percha and caoutchouc, to combine with the corundum or emery and form a solid body. While for this use celluloid has many serious defects, and is no better than shellac, caoutchouc has been found to possess many advantages over both. Wheels of all sizes, from one inch to forty-eight inches in diameter, and from one-fourth inch to six inches in thickness, made from caoutchouc combined with emery, have been used in the arts and manufactures for over twenty years. These wheels, particularly adapted for use in the dental laboratory, are superior to those which are in general use, because they cut faster and last longer. Yet I have not heard of any dentist that uses them, nor of any dental depot that keeps them in stock. Why the dental depots fail to keep these wheels I cannot tell. I simply know that if we depend upon the dental depots for *all* our supplies, we deprive ourselves of many useful implements. This will not be so if we simply look about us and note what we can advantageously appropriate from those used in the arts and manufactures.

Hard rubber and corundum disks, for use in the dental engine, can be obtained at the dental depots, but I have not been able to find stump-wheels made from the same material. The process of making these disks and wheels is a simple one, and every dentist has in his laboratory all the apparatus necessary for the purpose.

In offering the results of my experiments in this direction I may not present anything new, but as the method I now use is very different from my first attempt, and as I have reached it by gaining a step at a time, taking hints from here and there, it may be of interest and profit to others if I recount it to you in detail. When I commenced my experiments, about five years ago, I started with the idea that it was necessary to have, for the disks and wheels, a mould into which the rubber and corundum must be pressed, and while in the mould vulcanized. Finding this a slow and tedious process, I began experimenting, and finally adopted the very simple method which I present to you to-day.

The most difficult part, which is also the first step in the process, is combining the caoutchouc and corundum or emery. Taking a sheet of black rubber, so called, such as we use for making plates, I soften it over a water bath, not by dipping in hot water, as it must be worked dry. When softened, one side is covered with the emery (No. 90 if for cutting, No. 100 or 120 for polishing), then folded over on itself, and passed through a pair of rollers [a common clothes-wringer answers the purpose well], and this operation repeated until the proper quantity of emery is worked into the rubber. Use four parts of emery or corundum to one part of rubber, by weight. You observe that the combination of the rubber and emery is not a chemical, but simply a mechanical one.

This very tedious process would probably deter many from attempting to make these disks and wheels for themselves, but this objection has been removed, as at my solicitation The Keller Medicine Company, of Fort Wayne, Indiana, have already made arrangements to place the corundum-rubber on the market, and from a sample which they have sent me, I shall vulcanize a few disks and wheels before you to-day. After the corundum and rubber is properly combined, laying the rubber on a glass slab, and with a roller made by filling a bottle with boiling water, roll it to the thickness necessary for the disks and wheels,* and with the same cutters used for cutting sand-paper disks, cut out the disks or wheels. From scraps from the tin-shop I cut out disks of tin-plate somewhat larger, and stringing the corundum-rubber disks and tin-plates alternately on a wire the size of the screw of the mandrel used in the engine, clamp them together. For this purpose I use two square pieces of brass plate about one-eighth inch in thickness, with four holes in each for as many bolts, and screw them only tight enough to make a little pressure. They are now ready for the vulcanizer, and are vulcanized the same as a dental plate. As soon as removed from the vulcanizer, drawing out the wire, as it is more easily removed when hot, cool as rapidly as you wish, and when cold there is no trouble in separating the disks.

If it is desired to have hubs on the disks, take a number of pieces of brass plate, say about three-sixteenths of an inch thick, drill the hole for the wire, and countersink each side the size you wish the hub. By countersinking both sides you will need only half as many brass pieces, as the tin-plate can be used on the flat sides of

* To prevent the corundum-rubber adhering to the roller, dust a little of the corundum or emery over the surface.

the disks. To prevent them adhering to the brass, place a piece of tin foil between the disk and brass.

After the disks and wheels are vulcanized, true them up by placing them on the mandrel in the engine, and while running it, warm the edge of the disk in a flame of gas or alcohol till it softens, then run it against a piece of glass or porcelain.

To make the stub-wheels roll thicker than for the disks. Disks and wheels of different thickness can be vulcanized at the same time, but to vulcanize disks or wheels of different diameters it will be necessary to use heavier plates between them. Large wheels for laboratory use can be made in the same way as the small ones.

Disks and wheels for polishing fillings and teeth can also be made from vulcanite or soft rubber, and as the rubber for dental plates becomes hard rubber when vulcanized, it will be necessary to have a special rubber, one containing less sulphur, for this purpose, as the amount of sulphur added to the caoutchouc, and the length of time vulcanized, constitute the difference, after vulcanization, between hard rubber and vulcanite or soft rubber.

